# Applications of Machine Learning-Driven Molecular Models for Advancing Ophthalmic Precision Medicine

**DOI:** 10.3390/bioengineering12020156

**Published:** 2025-02-06

**Authors:** Rahul Kumar, Joshua Ong, Ethan Waisberg, Ryung Lee, Tuan Nguyen, Phani Paladugu, Maria Chiara Rivolta, Chirag Gowda, John Vincent Janin, Jeremy Saintyl, Dylan Amiri, Ansh Gosain, Ram Jagadeesan

**Affiliations:** 1 Department of Biochemistry and Molecular Biology, Miller School of Medicine, University of Miami, Miami, FL 33136, USA; rxk641@miami.edu (R.K.); gowdachirag24@gmail.com (C.G.); jxj2091@miami.edu (J.V.J.); anshgosain2009@gmail.com (A.G.); 2 Department of Ophthalmology and Visual Sciences, University of Michigan Kellogg Eye Center, Ann Arbor, MI 48105, USA; 3 Department of Clinical Neurosciences, University of Cambridge, Cambridge CB2 3EB, UK; ew690@cam.ac.uk; 4 Touro College of Osteopathic Medicine, New York, NY 10027, USA; rlee19@student.touro.edu; 5 Weill Cornell/Rockefeller/Sloan-Kettering Tri-Institutional MD-PhD Program, New York, NY 10065, USA; tun2001@med.cornell.edu; 6 Sidney Kimmel Medical College, Thomas Jefferson University, Philadelphia, PA 19107, USA; phani.paladugu@students.jefferson.edu; 7 Brigham and Women’s Hospital, Harvard Medical School, Boston, MA 02115, USA; 8 Department of Ophthalmology, University of Eastern Piedmont “A. Avogadro”, Via Ettore Perrone, 18, 28100 Novara, Italy; m.rivolta3@studenti.unisr.it; 9 Department of Chemistry, University of Miami, Coral Gables, FL 33146, USA; jjs1995@miami.edu; 10 Department of Biology, University of Miami, Coral Gables, FL 33146, USA; daa250@miami.edu; 11 Mecklenburg Neurology Group, Charlotte, NC 28211, USA; 12 Department of Computer Science, Whiting School of Engineering, Johns Hopkins University, Baltimore, MD 21218, USA; ramjagad@cisco.com

**Keywords:** precision medicine, machine learning in healthcare, molecular dynamics, neural network models, AI in clinical diagnostics

## Abstract

Ophthalmic diseases such as glaucoma, age-related macular degeneration (ARMD), and optic neuritis involve complex molecular and cellular disruptions that challenge current diagnostic and therapeutic approaches. Advanced artificial intelligence (AI) and machine learning (ML) models offer a novel lens to analyze these diseases by integrating diverse datasets, identifying patterns, and enabling precision medicine strategies. Over the past decade, applications of AI in ophthalmology have expanded from imaging-based diagnostics to molecular-level modeling, bridging critical gaps in understanding disease mechanisms. This paper systematically reviews the application of AI-driven methods, including reinforcement learning (RL), graph neural networks (GNNs), Bayesian inference, and generative adversarial networks (GANs), in the context of these ophthalmic conditions. RL models simulate transcription factor dynamics in hypoxic or inflammatory environments, offering insights into disrupted molecular pathways. GNNs map intricate molecular networks within affected tissues, identifying key inflammatory or degenerative drivers. Bayesian inference provides probabilistic models for predicting disease progression and response to therapies, while GANs generate synthetic datasets to explore therapeutic interventions. By contextualizing these AI tools within the broader framework of ophthalmic disease management, this review highlights their potential to transform diagnostic precision and therapeutic outcomes. Ultimately, this work underscores the need for continued interdisciplinary collaboration to harness AI’s potential in advancing the field of ophthalmology and improving patient care.

## 1. Introduction

**Figure 1:**
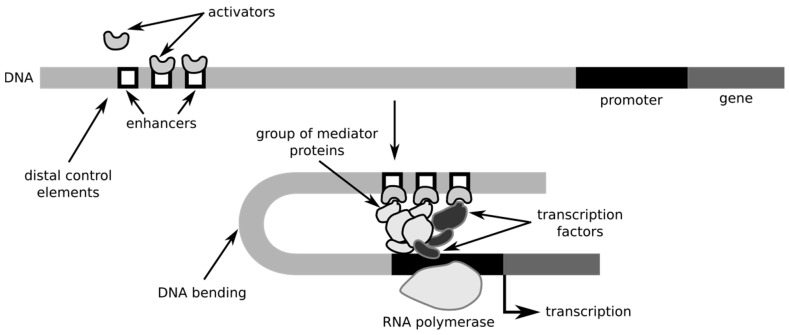
Mechanism of transcription factor-mediated gene activation. In the top section, TFs bind specific DNA enhancer sequences, targeting regulatory motifs with high TF-DNA affinity. This selective binding ensures transcriptional activation at precise loci. The lower section illustrates the recruitment of RNA polymerase II by bound TFs, which reshape DNA, enabling the polymerase to anchor to the promoter site, initiating mRNA synthesis. This TF-mediated stabilization is essential for transcription and cellular homeostasis, especially in ocular and neural tissues. Disrupted TF binding, as seen in ischemic optic neuropathy and optic neuritis, can impair gene regulation, leading to oxidative stress, inflammation, and neurodegeneration, clinically manifesting as progressive vision impairment. Reprinted with permission from Philippe Hupé, Wikimedia Commons under the Creative Commons Attribution-Share Alike 3.0 Unported License [3].

Molecular flux (MF) refers to the movement and interaction of molecules, such as proteins, lipids, and nucleic acids, within tissues, influencing gene regulation, cell signaling, and tissue homeostasis. In ocular and neural tissues, disruptions in molecular flux are central to the pathogenesis of various optic neuropathies, including ischemic optic neuropathy (ION), optic neuritis, glaucoma, traumatic optic neuropathy, and Leber’s hereditary optic neuropathy (LHON), among others. In these conditions, altered molecular dynamics can impair vital processes such as gene expression, immune responses, and cellular repair mechanisms, leading to optic nerve damage and vision loss. For example, in ischemic optic neuropathy, hypoxia-induced disruptions in molecular flux result in endothelial dysfunction and inflammatory cytokine release, while in optic neuritis, autoimmune responses and cytokine dysregulation contribute to axonal damage and demyelination. Similarly, in diseases like glaucoma and diabetic optic neuropathy, increased intraocular pressure and metabolic disturbances, respectively, disturb cellular signaling pathways, exacerbating retinal ganglion cell death (Figure 1). Furthermore, inherited disorders like LHON and retinitis pigmentosa highlight how mutations in specific genes can alter the molecular environment, triggering neurodegeneration [1,2]. Understanding how molecular flux governs these disease processes and leads to optic nerve damage is crucial for developing targeted therapies that can preserve vision and improve patient outcomes.

Advanced AI and machine learning (ML) technologies are now allowing us to model these molecular interactions with unprecedented precision, providing insights directly applicable to ophthalmic conditions. For example, reinforcement learning (RL) simulates agent-based molecular behavior [4], helping us track TF binding efficiency and interaction patterns in ischemic or inflammatory settings. In ischemic optic neuropathy, RL can model how hypoxic environments affect TF navigation, highlighting pathways vulnerable to oxygen deprivation that contribute to neural damage. Graph neural networks (GNNs) further enhance our understanding by constructing molecular interaction networks within diseased neural tissues [5]. GNNs allow us to map interactions between TFs, enzymes, and other molecules, particularly in contexts where competitive binding and altered affinity influence disease progression. This approach may be valuable in identifying molecular interactions disrupted by increased intracranial pressure, ischemia, demyelination, or inflammation, as seen in various optic neuropathies [6].

In settings where molecular environments are unpredictable, Bayesian inference models provide probabilistic predictions of molecular behavior [7], accommodating the variability in tissue composition and molecular concentrations seen in diseases such as glaucoma or age-related macular degeneration (ARMD). Bayesian models allow clinicians to target the most probable pathologic interactions, such as those driving inflammatory damage in optic neuritis, thus refining treatment approaches. Finally, generative models like generative adversarial networks (GANs) create synthetic datasets that replicate the MF [8]. By simulating altered molecular dynamics under conditions like hypoxia or inflammation, GANs provide extensive experimental data, revealing early molecular changes that could serve as intervention points.

From this perspective, we explore the potential of AI and ML techniques to transform the understanding and treatment of specific ophthalmic diseases, including glaucoma and age-related macular degeneration (ARMD). By bridging molecular biology with advanced computational models, these technologies can inform precision medicine, offering clinicians tools to predict disease progression and tailor therapies more effectively for conditions impacting vision and neural health. However, to avoid bias and realistically represent the complex, multi-dimensional nature of these diseases, it is essential that AI and ML models are designed to incorporate diverse data sources and account for the varying biological and clinical factors that influence disease outcomes. This requires careful attention to data quality, ensuring that training datasets reflect a broad range of patient demographics, disease stages, and environmental variables. Furthermore, models should be validated across different populations and clinical settings to ensure robustness and generalizability. Incorporating multiple levels of modeling—from molecular pathways to clinical outcomes—can help mitigate bias by offering a more holistic view of disease progression, enhancing the precision and applicability of predictive models. By developing these nuanced, multi-level models, we can achieve a deeper understanding of disease mechanisms and optimize personalized treatment strategies, ultimately improving patient outcomes in both glaucoma and ARMD.

## 2. Molecular Movements Driving Health and Disease

MF—the regulated movement of molecules across cellular environments—plays a pivotal role in maintaining the functionality of optic nerve and retinal tissues, especially in relation to the diffusion of signaling molecules and transcription factors (TFs). Mathematically, MF is governed by Fick’s First Law, expressed as J = −D(ΔC/Δx), where the flux J is determined by the diffusion coefficient D (a measure of how easily molecules move through a medium) and the concentration gradient ΔC/Δx (the rate of change in concentration over distance). In the context of the optic nerve, this process facilitates the precise delivery of TFs to critical genomic regions. Under normal conditions, the flux of TFs allows them to bind to DNA enhancer sequences, effectively modulating gene expression necessary for maintaining cellular homeostasis, synaptic function, and tissue integrity. For instance, TFs like NF-κB and HIF-1α are crucial for regulating inflammation and the response to hypoxia within retinal ganglion cells (RGCs), ensuring the proper cellular response to environmental cues. However, when molecular flux is disrupted, such as in diseases like ischemic optic neuropathy or optic neuritis, the delivery and interaction of these TFs become impaired. This can prevent the proper activation of genes involved in neuronal survival and repair, exacerbating neurodegenerative processes and contributing to vision loss. Similarly, disruptions in TF localization and binding can lead to faulty transcriptional regulation, creating an environment conducive to inflammation, oxidative stress, and cellular apoptosis, all hallmarks of optic nerve pathology. In clinical settings, understanding these disruptions in molecular flux can provide insights into the underlying mechanisms of optic neuropathies and offer potential therapeutic targets for intervention.

In glaucoma, elevated intraocular pressure (IOP) disrupts the eye’s fluid dynamics, generating mechanical stress and often reducing blood flow within the optic nerve head. This dual impact—mechanical and vascular—triggers a cascade of molecular events that contribute to cellular damage across multiple phases of the disease. In the pre-clinical phase, there is an increase in intraocular pressure that begins to stress the optic nerve fibers, leading to early disruptions in axoplasmic flow, which affects retinal ganglion cell (RGC) function and survival. In the early phase, mechanical stress on the optic nerve head and reduced blood flow lead to ischemic conditions and oxidative stress, initiating mitochondrial dysfunction in RGCs. These early molecular disruptions impair cellular metabolism and increase the production of reactive oxygen species (ROS), which accelerate cell damage. In the late phase, this persistent oxidative stress, along with chronic ischemia, leads to the irreversible apoptosis of RGCs and the thinning of the retinal nerve fiber layer (RNFL), a hallmark of glaucoma. Moreover, in the immune phase, inflammatory cytokines and glial cell activation play a key role in neuroinflammation, further exacerbating RGC degeneration. The cornea and ocular surface are also impacted in glaucoma, with the anterior segment of the eye showing changes that reflect the underlying disease processes in the posterior segment. The corneal endothelium, which is crucial for maintaining corneal clarity, may exhibit stress-induced alterations, such as a reduction in endothelial cell density, which can be visualized using specular microscopy. These early signs of cellular stress in the corneal endothelium may serve as potential biomarkers for early glaucomatous changes, preceding noticeable optic nerve damage. Together, these phase-specific mechanisms highlight the complex, multi-phase nature of glaucoma and the importance of understanding how different cellular components contribute to disease progression at each stage.

Compromised mitochondrial efficiency diminishes ATP availability, weakening transcription factor (TF) activity and limiting the activation of protective gene networks that shield RGCs from oxidative damage and cellular dysfunction [9]. This metabolic dysregulation extends to the corneal epithelium, where altered gene expression profiles may be detectable through advanced molecular techniques such as RNA sequencing of corneal impression cytology samples. Oxidative stress can also directly disrupt TF binding to DNA, further impairing essential gene regulation for RGC survival [10]. These molecular perturbations may manifest as subtle changes in corneal nerve density and morphology, detectable through in vivo confocal microscopy (IVCM), offering a potential non-invasive biomarker for glaucoma progression.

Clinically, glaucoma often manifests as gradual peripheral vision loss, typically unnoticed until the disease has advanced significantly. Optic disk cupping, observable during examinations, indicates ongoing RGC loss and remodeling of the optic nerve rather than simply reflecting apoptosis [9]. Advanced imaging techniques like optical coherence tomography angiography (OCTA) can reveal microvascular changes in both the retina and the peripapillary region, potentially correlating with alterations in corneal vasculature at the limbus. Machine learning (ML) models, leveraging multi-modal imaging data, including corneal topography and pachymetry, could potentially identify subtle structural changes associated with glaucomatous damage before conventional clinical signs become apparent.

When left untreated, these molecular disruptions and impaired TF function drive a cascade, leading to progressive vision loss and, ultimately, potential blindness [9]. The cornea, as the eye’s first refractive surface, may undergo biomechanical changes in response to elevated IOP, detectable through technologies like Scheimpflug imaging or dynamic corneal response analysis. These corneal biomechanical properties could serve as additional parameters in ML-driven predictive models for glaucoma progression.

Similarly, in antibody-mediated optic neuritis (e.g., myelin oligodendrocytic glycoprotein (MOG) or aquaporin-4 (AQP4) neuromyelitis optica spectrum disorder), inflammatory cytokines precipitate cellular congestion, hampering molecular traffic across the optic nerve head [11]. Here, flux dynamics are altered as edema and immune infiltration disrupt TF mobility and binding efficiencies, skewing intracellular signaling pathways crucial for optic nerve resilience. This inflammation results in the accumulation of reactive oxygen species (ROS), further damaging cellular architecture [12]. The inflammatory cascade may extend to the anterior segment, potentially manifesting as subtle changes in corneal thickness or curvature, detectable through high-resolution anterior segment OCT or Scheimpflug imaging.

At the bedside, patients present with severe, sometimes bilateral, vision loss and retro-orbital pain and typically demonstrate optic disk edema on fundoscopic examination [12]. In progressive cases, chronic inflammation can lead to optic atrophy, irreversible visual loss, and sustained neurodegenerative burden [11]. Advanced ML algorithms, integrating data from corneal confocal microscopy, tear film biomarkers, and conventional ophthalmic imaging, could potentially identify subclinical inflammatory changes in the cornea and ocular surface associated with optic neuritis. This approach might enable earlier diagnosis and treatment initiation, potentially mitigating long-term visual outcomes.

Both antibody-mediated optic neuritis and glaucoma involve MF disruptions that impair TF mobilization and binding fidelity, weakening gene regulatory functions critical for optic nerve health. Clinically, these impairments underscore the need for early, targeted interventions to prevent irreversible damage. In optic neuritis, immunomodulatory therapies are essential for controlling inflammation and preserving optic nerve function [6], while glaucoma therapies primarily focus on reducing IOP and supporting ocular blood flow to minimize oxidative damage [9,10]. Addressing MF dysfunction and enhancing TF availability could be pivotal in sustaining vision and mitigating neurodegenerative progression in these optic nerve disorders.

## 3. Leveraging AI to Map and Correct MF Disruptions

I. Reinforcement Learning (RL): RL models can simulate TFs as active agents navigating the cellular environment, accounting for molecular and metabolic disturbances like ROS elevation and adenosine triphosphate (ATP) scarcity that alter binding efficiency [13]. By structuring RL models with rewards linked to TF binding at precise genomic locations [14], these systems simulate TFs’ molecular paths within hypoxic tissue, where navigation pathways are hindered by reduced ATP. RL thus identifies key metabolic chokepoints, such as regions with the greatest ATP depletion and highest ROS density, which most significantly disrupt TF-DNA binding.

Clinically, these insights might guide ophthalmologists on when and where to implement targeted therapeutic interventions. For example, RL-based models may reveal hypoxic zones where TF binding efficiency drops below a critical threshold. Clinicians could potentially use these data to prioritize hyperbaric oxygen or vascular treatments aimed specifically at restoring ATP balance in those hypoxic regions [15], thus stabilizing TF binding and preserving optic nerve function. Furthermore, RL simulations can identify hypoxia-sensitive pathways within TF networks, informing gene therapy approaches designed to bolster those pathways, effectively delaying or preventing optic neuropathy progression.

II. Graph Neural Networks (GNNs): GNNs model MF by creating dynamic maps of molecular interactions within an inflamed optic nerve environment, where cytokines and immune cell traffic obstruct normal cellular diffusion [16]. In antibody-mediated optic neuritis, immune infiltration leads to altered diffusion rates and skewed molecular binding affinities, impacting the MF of essential TFs and enzymes. By weighting edges within GNNs according to binding probabilities and cytokine influence on specific nodes (e.g., TFs or enzymes vital for myelin stability), GNNs reveal critical vulnerabilities within molecular pathways [17].

Clinically, GNNs might allow for ophthalmologists to visualize specific molecular networks within the optic nerve affected by inflammation, identifying pathways with the highest cytokine-induced disruption. For instance, GNN models may highlight that TNF-alpha-mediated pathways show reduced TF activity at myelin-protective genes [18], suggesting a need for TNF-alpha inhibitors to preserve optic nerve function. This approach enables the more precise selection of anti-inflammatory treatments and helps prioritize agents that will directly impact disrupted molecular pathways, offering a more targeted approach to MOG optic neuritis management and improving patient outcomes by reducing optic nerve atrophy.

III. Bayesian Inference: Bayesian inference models integrate prior MF data with real-time patient data to provide probabilistic predictions on TF binding disruptions and enzyme inactivation under varied clinical conditions [19], such as ischemic episodes or inflammation spikes. By incorporating variability in TF concentrations, cytokine levels, and metabolic stressors, Bayesian models predict the most likely MF disruptions in a given clinical state, helping clinicians anticipate molecular dysfunctions even as patient conditions evolve.

These probabilistic models may allow clinicians to proactively address expected disruptions. For example, if a Bayesian model forecasts high probabilities for TF displacement due to cytokine surges in optic neuritis [20], clinicians can pre-emptively administer cytokine-targeted therapies to mitigate the predicted disruption. Similarly, if Bayesian inference predicts elevated chances of oxidative damage to critical TF pathways in ischemic optic neuropathy [21], clinicians can prioritize antioxidants that fortify those pathways, ultimately stabilizing gene expression essential for optic nerve function. This predictive approach can tailor interventions to real-time molecular needs, enhancing the precision of clinical treatments and improving patient outcomes.

IV. Generative Adversarial Networks (GANs): GANs create synthetic datasets that can model MF disruptions under controlled and predicted variations in hypoxia, ROS levels, and cytokine activity [8,22], providing extensive experimental insights into disease-specific molecular dynamics. For example, in ischemic optic neuropathy, GANs can simulate fluctuating oxygen levels and their impact on TF binding [6], generating data on how hypoxia impedes flux at different time points. By iterative training using real data, GANs can mimic molecular responses under various therapeutic conditions, showing how incremental changes in oxygenation or cytokine levels alter TF efficiency and gene regulatory functions.

Clinicians benefit from GAN-based simulations by gaining data-driven insights into the most responsive molecular targets within ocular tissue. For instance, GANs might simulate the effectiveness of antioxidants across different ROS levels, revealing an optimal threshold for therapeutic interventions in ischemic conditions [23]. By providing synthetic, pathology-specific data, GANs allow clinicians to refine treatment plans with unprecedented granularity, enabling them to anticipate molecular disruptions before they reach irreversible stages. Additionally, GAN-simulated datasets can help ophthalmologists identify early intervention points, such as specific TF pathways that, if stabilized early, could prevent downstream neurodegeneration and vision loss in optic neuropathies (Table 1).

Building on this, the integration of these models highlights their transformative potential for molecular flux modeling and neuro-ophthalmic pathology. For instance, Bayesian inference supports probabilistic modeling by accommodating variability in tissue composition and molecular concentrations, refining the identification of pathologic interactions in diseases such as glaucoma and ARMD. GANs enhance this by generating synthetic datasets, simulating altered molecular dynamics under conditions like hypoxia or inflammation, and revealing early intervention points. RL’s ability to predict cytokine surges and optimize therapeutic timing, coupled with GNNs’ capacity to map cytokine-driven disruptions, enables personalized treatment strategies with unprecedented precision.

The true novelty lies in the practical application of these models and the insights they offer. For example, integrating molecular flux data with imaging techniques like OCT and fMRI could provide dynamic insights into disease progression, bridging molecular-level disruptions with clinical markers like RNFL thinning. However, to fully establish innovation, this approach must demonstrate how these combinations surpass existing methods, address previously unsolved challenges, and uncover novel insights into complex diseases like ischemic optic neuropathy, MOG optic neuritis, glaucoma, and ARMD. Together, these interdisciplinary approaches promise to revolutionize precision medicine, offering clinicians actionable tools to optimize outcomes and improve patient care in neuro-ophthalmic diseases.

## 4. Integrated AI-Enhanced Approach to MF Management: From Predictive Analytics to Personalized Clinical Applications

Each AI model—RL for pathway vulnerability in ischemic conditions, GNNs for inflammatory network mapping, Bayesian models for probabilistic predictions, and GANs for therapeutic simulations—provides distinct insights into MF, collectively forming a robust, integrated framework for clinical decision-making. When combined, these technologies offer a comprehensive view of molecular dynamics, allowing clinicians to visualize, predict, and proactively address disruptions in MF specific to each patient’s neuro-ophthalmic condition.

For example, in a patient with progressive optic neuritis, the combined AI approach might allow clinicians to visualize cytokine-mediated disruptions (using GNNs) [16,17], anticipate TF displacement under worsening inflammation (through Bayesian models) [19], and simulate the effects of specific anti-inflammatory therapies (with GANs) [22,23]. Similarly, in ischemic optic neuropathy, RL can identify critical points of TF binding failure, GNNs can map compensatory pathways, and GANs can simulate the protective effects of incremental oxygenation therapies. Together, these insights support an adaptive, real-time response to MF alterations, allowing ophthalmologists to make informed treatment adjustments based on specific molecular disruptions as they arise. Through this multi-layered AI-driven framework, clinicians gain the ability to approach neuro-ophthalmic diseases dynamically, treating conditions as evolving processes with specific molecular markers. This enables a more precise and patient-specific approach to managing vision-threatening diseases, improving clinical outcomes by preserving the molecular structures that support optic nerve and retinal health.

## 5. Discussion

Despite significant strides in using AI and ML to model molecular framework (MF) disruptions, there are ongoing limitations and areas of debate regarding the precision, utility, and feasibility of these approaches in clinical practice. One prominent limitation lies in the models’ inability to fully capture the complex interplay of molecular actors—including transcription factors (TFs), inflammatory cytokines, and metabolic intermediates—in dynamic disease environments such as ischemic optic neuropathy and optic neuritis [24,25,26]. While current algorithms excel at identifying general patterns, their predictive power is often constrained by the variability in individual MF profiles, shaped by genetic predispositions, epigenetic modifications, and metabolic states [27,28].

This variability underscores the need for more personalized MF data integration, involving the incorporation of multi-omics data (genomic, transcriptomic, and proteomic) to capture patient-specific signatures [29,30]. However, significant barriers remain, including the challenge of integrating diverse datasets, the scarcity of large, well-annotated cohorts, and the computational complexity of such approaches [31,32]. Moreover, the feasibility of translating these advancements into clinical settings raises questions about resource allocation, regulatory approval, and clinician adoption [33,34,35,36]. Addressing these issues will require not only technical innovation but also interdisciplinary collaboration to ensure that these tools reach their full potential in advancing personalized medicine.

### 5.1. Data and Computational Constraints

The lack of high-resolution, time series MF data is a limitation for training precise models. Currently, AI models often rely on static or sparse datasets that fail to capture real-time changes in MF, especially in fluctuating pathological states like the hypoxia-induced TF binding instability observed in ischemic optic neuropathy [25]. To enhance data granularity in tracking disease progression, integrating continuous in vivo molecular sampling and advanced imaging modalities could yield high-resolution datasets that more accurately capture temporal molecular dynamics and reflect intricate pathological shifts at each disease stage [26]. Real-time data from optic nerve head sampling, for example, could be especially valuable for GNNs designed to map interactive pathways under high inflammatory conditions [27]. The integration of such data could theoretically improve the model’s predictive capability in fluctuating environments but would require computational infrastructure capable of handling high-throughput, time series data.

Advancing parallel processing and quantum computing is essential to meet the computational demands of RL and GNNs in flux analysis. RL models simulate numerous potential TF pathways within disrupted molecular environments, such as ATP-depleted and oxidative stress regions in ischemic optic neuropathy [20]. Quantum computing could theoretically expand RL’s capacity to process diverse pathways concurrently, enhancing model fidelity and accelerating TF binding disruption simulations. However, limited quantum resource access in healthcare [28], along with significant infrastructure and cost requirements, currently constrains the clinical implementation of these technologies.

### 5.2. Model Interpretability and Clinical Translation

Another ongoing debate in the field concerns the interpretability of these complex AI models, as clinicians require actionable outputs to make precise interventions [29]. Reinforcement learning models that highlight hypoxia-sensitive TF pathways or GNNs that map cytokine-driven disruptions provide critical insights, but translating these outputs into clinical recommendations is challenging. Most current models lack the transparency necessary for clinicians to confidently apply their findings in patient care. For example, in cases of MOG optic neuritis, RL-based outputs indicating regions of TF binding inhibition due to cytokine-mediated congestion could inform treatment strategies, such as localized anti-inflammatory delivery. Yet, clinicians often lack direct interpretability tools, like pathway visualizations, that explicitly show how these molecular disruptions correlate with observed clinical symptoms such as optic nerve swelling or retro-orbital pain.

Future model architectures should prioritize interpretability enhancements, such as graph-based visualization tools within GNNs that allow clinicians to view TF pathway disruptions in relation to clinical imaging data, including optic disk edema or retinal nerve fiber layer (RNFL) thickness changes [30]. Moreover, by combining these visualizations with patient-specific molecular data, such models could theoretically enable more precise treatment regimens. Clinical decision–support interfaces are being explored to bridge this gap by creating user-friendly tools that interpret model data in real time, but these tools require further validation to ensure accuracy and usability in diverse clinical settings.

### 5.3. Ethical and Regulatory Considerations

The development and application of AI in MF modeling raise potential ethical and regulatory questions, particularly regarding patient data privacy, model validation, and the potential for biases within predictive models. Since MF varies significantly across different populations and even individual patients, there is a risk that AI models could perpetuate biases if they are trained predominantly on data from specific demographic groups. In diseases like glaucoma, where molecular disruptions can present uniquely based on underlying systemic health issues [10,27], ensuring that model training datasets encompass diverse patient profiles is critical to avoiding disparities in care.

Furthermore, the field faces regulatory challenges in validating these AI models for clinical use, as the current frameworks often fall short in addressing the complexity and novelty of AI-driven molecular simulations. To bridge this gap, future AI models could benefit from frameworks similar to those used in pharmacogenomics, which consider patient-specific data variability and aim for FDA or EMA approval standards specifically tailored to adaptive AI models [31]. Regulatory bodies are encouraged to develop standards that balance innovation with rigorous safety and efficacy benchmarks, fostering the clinical translation of these technologies while ensuring patient safety.

## 6. Conclusions

AI-driven MF modeling has the potential to revolutionize the clinical landscape of disease management. Through reinforcement learning, Bayesian inference, GANs, and GNNs, clinicians gain insights into molecular dynamics, offering precise interventions in real time. By simulating molecular disruptions in ischemic optic neuropathy, optic neuritis, and other vision-related conditions, AI models guide treatment decisions, from TF pathway restoration in hypoxic environments to inflammation-targeted therapeutics in optic neuritis. As the field advances, challenges persist, including data resolution and model interpretability. Overcoming these will require enhanced computational infrastructure, interdisciplinary collaboration, and patient-centered regulatory frameworks, ensuring the safe integration of these transformative technologies. This fusion of AI with precision medicine marks a promising trajectory in neuro-ophthalmology, improving disease outcomes and expanding the scope of personalized care.

## 7. Future Directions and Integrative Approaches

The future of AI-driven molecular flux (MF) modeling lies in integrating multiple machine learning (ML) modalities to capture a more comprehensive view of neuro-ophthalmic pathology. For example, combining Bayesian inference, generative adversarial networks (GANs), and reinforcement learning (RL) can model the probabilistic dynamics of molecular flux under varied clinical conditions while generating synthetic datasets to test new therapeutic interventions [37,38,39,40].

Although plasmapheresis and intravenous immunoglobulin (IVIG) remain foundational for managing MOG optic neuritis [41,42,43,44,45], advanced ML models—like RL and graph neural networks (GNNs)—offer insights into molecular network vulnerabilities. RL enables clinicians to predict transcription factor (TF) binding disruptions in inflammatory zones, helping pinpoint cytokine surges that could destabilize key molecular pathways within the optic nerve [46,47,48,49,50]. This allows for the precision timing of interventions, such as anti-cytokine therapies or IVIG administration, to preserve neural integrity before inflammation worsens [51,52]. Similarly, GNNs provide a detailed map of cytokine-driven disruptions, visualizing molecular dynamics in cytokine-affected optic nerve tissues. This capacity can guide personalized treatment regimens, optimizing plasmapheresis and IVIG applications based on predictive inflammation markers. Such AI-driven insights can refine therapeutic timing, predict neurodegenerative changes, and ultimately enhance care for MOG optic neuritis [53].

Future models may also integrate multi-modal data sources—combining MF data with advanced imaging techniques like optical coherence tomography (OCT) and functional magnetic resonance imaging (fMRI)—to generate dynamic insights into disease progression [54]. This could support more precise diagnoses and therapeutic adjustments by linking molecular disruptions to real-time imaging markers, such as retinal nerve fiber layer (RNFL) thinning or optic disk pallor in ischemic optic neuropathy [55]. Achieving this vision will require advancements in data interoperability to allow AI models to synthesize complex data streams, a challenge that remains technically demanding [56].

In summary, while AI-driven MF modeling has transformative potential for neuro-ophthalmic diseases, challenges such as data limitations, computational demands, interpretability, and regulatory concerns must be addressed [57,58]. Technological innovations, interdisciplinary collaborations, and rigorous validation processes are essential to empower clinicians with real-time, precise insights into molecular disruptions, enabling more effective and personalized patient care.

## Figures and Tables

**Table 1:** Overview of ML models for clinical applications in disease management. This table provides a concise summary of the capabilities and applications of various AI and ML models—reinforcement learning (RL), graph neural networks (GNNs), Bayesian inference, and generative adversarial networks (GANs)—in the context of neuro-ophthalmic disease. Each model is described with respect to its function in modeling MF and its specific clinical implications, such as identifying therapeutic targets, mapping inflammatory pathways, predicting molecular disruptions, and simulating therapeutic outcomes. This organized overview serves as a practical guide for clinicians seeking to integrate these technologies into precision medicine approaches to address complex pathologies like ischemic optic neuropathy and MOG optic neuritis.

ML Model	Primary Function	Clinical Utility	Specific Applications
RL	Simulates TF navigation paths in hypoxic environments, identifying key metabolic chokepoints.	Assists in prioritizing interventions like hyperbaric oxygen therapy and gene therapy to enhance ATP balance in hypoxic regions.	Useful in ischemic optic neuropathy to determine when ATP restoration therapies can stabilize TF-DNA binding.
GNNs	Maps molecular interactions within inflamed optic nerve environments, highlighting areas of disrupted MF.	Identifies cytokine-induced molecular vulnerabilities, enabling targeted anti-inflammatory treatment selection in optic neuritis.	Applies to MOG optic neuritis in order to prioritize treatments that counteract TNF-alpha-mediated disruptions to myelin-protective genes.
Bayesian Inference	Provides probabilistic predictions on TF binding disruptions and enzyme inactivation under varying clinical conditions.	Forecasts molecular disruptions to guide early intervention with targeted therapies, optimizing treatment precision.	Provides real-time adaptive treatment strategies for inflammatory surges in neuro-ophthalmic diseases.
GANs	Generates synthetic datasets to model MF disruptions, providing insights into early intervention points.	Reveals optimal therapeutic thresholds, aiding in preemptive intervention to stabilize molecular pathways in optic neuropathies.	Simulates effects of ROS levels on TF efficiency, aiding in antioxidant treatment planning for ischemic conditions.
